# Mean platelet volume/platelet count ratio in colorectal cancer: a retrospective clinical study

**DOI:** 10.1186/s12885-019-5504-9

**Published:** 2019-04-04

**Authors:** Yang-Yang Wu, Xuan Zhang, Yuan-Yuan Qin, Jin-Qiu Qin, Fa-Quan Lin

**Affiliations:** Department of Clinical Laboratory, the First Affiliated Hospital of Guangxi Medical University, Nanning, 530021 Guangxi Zhuang Autonomous Region China

**Keywords:** Mean platelet volume/platelet count ratio, Colorectal cancer, Adenomatous polyp

## Abstract

**Background:**

Mean platelet volume (MPV) is a marker of platelet activation. MPV and platelet count (PC) are negatively correlated, and their ratio (MPV/PC) is informative for the diagnosis of malignant tumors. However, the relationship between MPV/PC and colorectal cancer is unclear. This retrospective clinical study aimed to evaluate the diagnostic value of MPV/PC in colorectal cancer.

**Methods:**

Hematological examinations were performed at initial diagnosis in patients with colorectal cancer (*n* = 186) or adenomatous polyp (*n* = 132) and healthy controls (*n* = 108). Hematological parameters evaluated included white blood cells, red blood cells, hemoglobin, neutrophils, lymphocytes, monocytes, PC, and MPV. Statistical analyses included Student’s t-test, one-way ANOVA or Kruskal-Wallis H test, chi-square tests, Spearman’s correlation test and receiver operating characteristic (ROC). ROC curve was used to evaluate the diagnostic values of MPV and MPV/PC in colorectal cancer.

**Results:**

Among these groups, MPV was significantly lower in colorectal cancer than in adenomatous polyp (*p* = 0.002) and healthy controls (*p* < 0.001) but did not significantly differ between adenomatous polyp and healthy controls (*p* = 0.210). MPV/PC was lower in colorectal cancer compared with adenomatous polyp and healthy controls (*p* < 0.001) and in adenomatous polyp compared with healthy controls (*p* = 0.010). MPV did not significantly differ among colorectal cancer subgroups, while MPV/PC significantly differed between TNM stages and the presence/absence of lymph node metastasis. MPV/PC was negatively correlated with the neutrophil to lymphocyte ratio(NLR) (*p* = 0.002) and platelet to lymphocyte ratio(PLR) concentration (*p* < 0.001). In the differential diagnosis between colorectal cancer and adenomatous polyp, MPV/PC produced a larger ROC curve than MPV, NLR or PLR alone. Using MPV/PC to distinguish between colorectal cancer and controls produced a larger AUC than using MPV or NLR alone.

**Conclusions:**

MPV/PC may be useful for the diagnosis of colorectal cancer. However, further studies are warranted to include additional regions and more data, to assess the utility of MPV/PC as a novel diagnostic screening tool for colorectal cancer.

## Background

Colorectal cancer is the third most common malignant tumor and the fourth leading cause of cancer-related deaths worldwide [1]. In China, there were 376,300 new cases of colorectal cancer, and 191,000 cases died of colorectal cancer in 2015 [2]. It is estimated that there will be more than 1.8 million new colorectal cancer cases and 881,000 deaths worldwide in 2018 [3]. Colorectal cancer takes years or even decades to develop, and many people do not develop clinical manifestations such as abdominal pain or intestinal bleeding until cancer metastasizes [4, 5]. Although patients with colorectal cancer can be treated with a combination of surgery, chemotherapy, and radiotherapy, a high recurrence rate, and distant metastases still threaten a large proportion of patients [6]. At present, colonoscopy dramatically improves the diagnostic rate of colorectal cancer, but its application is limited by its invasiveness, high cost, and inconvenience of operation [4]. Therefore, it is necessary to find a sensitive and straightforward index for the diagnosis of colorectal cancer.

The mean platelet volume (MPV) reflects the size of platelets, which is related to platelet production and activation [7, 8]. Studies have found that MPV is closely related to many kinds of tumors, such as colon cancer, thyroid cancer, and renal cell carcinoma [7, 9, 10]. Additionally, it has been shown that there is an inverse relationship between MPV and platelet count (PC), suggesting that these two variables should be interpreted as a ratio rather than being used alone [11–13]. Both MPV and PC are commonly available because they are inexpensive and simple parameters of whole blood counts, which are used as routine examination items in outpatients and inpatients.

Recently, much attention has been directed to the clinical value of the MPV/PC ratio in malignant tumors including hepatocellular carcinoma, pancreatic cancer, lung cancer, and other diseases [14–16]. However, as far as we know, the relationship between MPV/PC and colorectal cancer has not been reported. The purpose of this study was to investigate the relationship between MPV/PC and the clinicopathological features of colorectal cancer and to evaluate its value for the diagnosis of colorectal cancer.

## Methods

### Study population

This study retrospectively analyzed the patients with colorectal cancer who were first diagnosed in the First Affiliated Hospital of Guangxi Medical University between June 2012 and May 2018. Patients who had undergone surgical resection after being diagnosed with colorectal cancer but did not receive pharmacological treatment were included in the study. The exclusion criteria were as follows: pregnancy or lactation, other malignancies, thyroid disease, diabetes, cardiovascular disease, autoimmune diseases (such as idiopathic thrombocytopenic purpura), kidney disease, hematological disease, or blood transfusion within 3 months before admission. One hundred and eighty-six patients with colorectal cancer were included in the study and all patients were staged according to the 7th edition of the United States Joint Cancer Committee (UICC/AJCC) TNM staging criteria. One hundred and thirty-two patients diagnosed with colorectal adenomatous polyp were assigned to the adenomatous polyp group. Simultaneously, one hundred and eight healthy subjects were selected as the control group. There was no significant difference in gender or age between the three groups. The study was approved by the Ethics Committee of the First Affiliated Hospital of Guangxi Medical University. All the participants gave written informed consent.

### Clinical measurements and calculation

Fasting venous blood was taken into an ethylenediaminetetraacetic acid-K_2_ anticoagulant tube and a dry tube early during the early morning. A Beckman 780 blood cell analyzer (Beckman Coulter, Brea, CA) was used for the routine examination of blood samples. The laboratory data included white blood cells, red blood cells, hemoglobin, platelets, neutrophils, lymphocytes, monocytes, and mean platelet volumes. MPV/PC values were calculated from the mean platelet volume and the platelet count.NLR values were calculated from the neutrophil and the lymphocyte. PLR values were calculated from the platelet count and the lymphocyte.

### Statistical analyses

The continuous variable data are expressed as mean ± standard deviation or median (interquartile range), and the categorical variable data are expressed in terms of frequency or rate. All data were statistically analyzed using the software programs SPSS 20.0 (IBM, Armonk, NY, USA), MedCalc 15.0 (MedCalc Software, Mariakerke, Belgium), and Prism 5 (GraphPad Software, San Diego, CA, USA). Data were compared between two groups by Student’s *t*-test. Data were compared between three groups by one-way ANOVA or Kruskal-Wallis H test. The Chi-square test was used for the comparison of rates. Correlations were analyzed by Spearman’s correlation test. The receiver operating characteristic (ROC) curve was used to calculate the sensitivity, specificity, positive predictive value, negative predictive value, and area under the curve (AUC), and to evaluate the diagnostic values of MPV and MPV/PC in colorectal cancer. Statistical significance was defined as *p* < 0.05.

## Results

### Clinical characteristics of the subjects

**Fig. 1:**
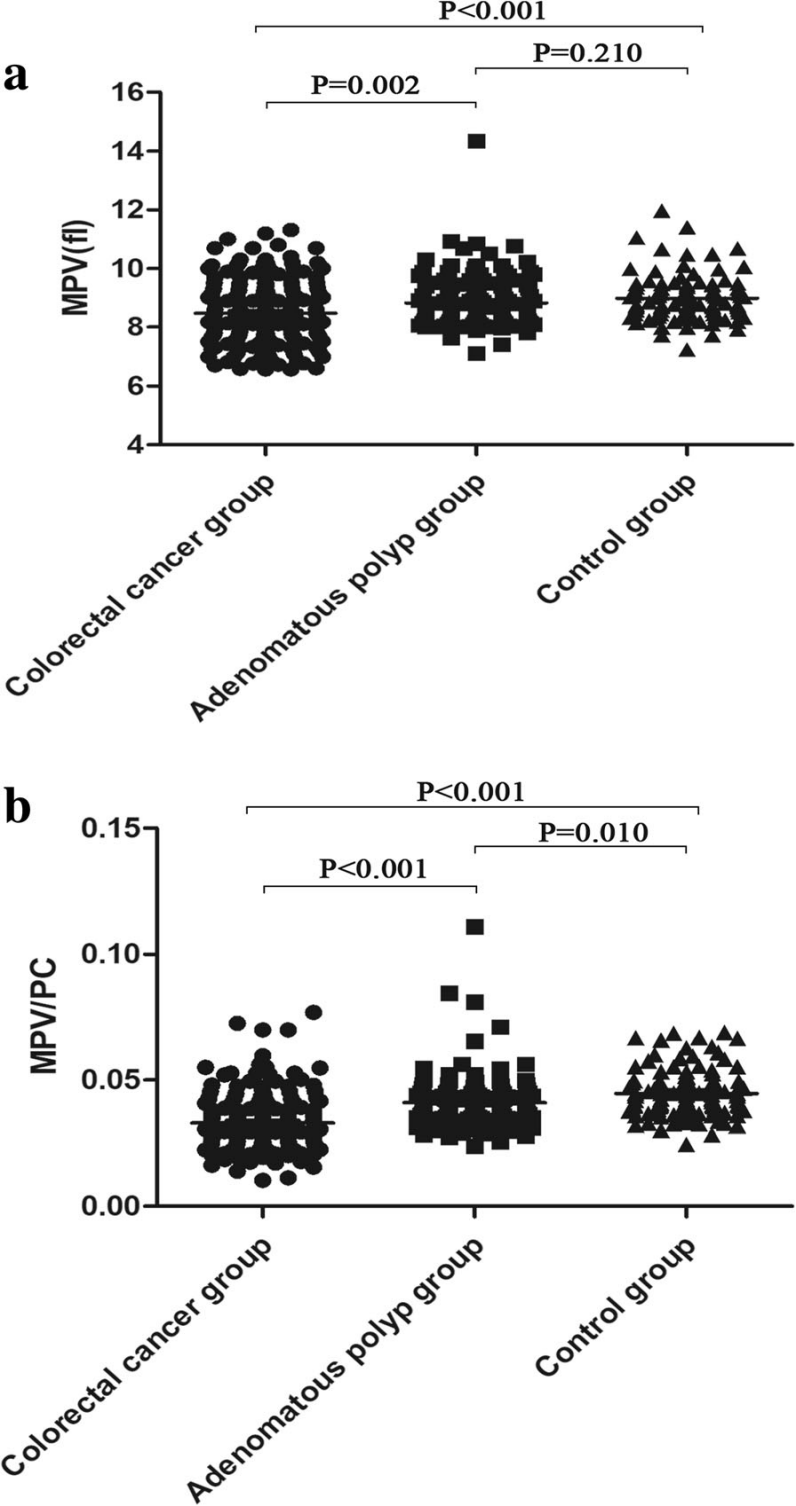
MPV and MPV/PC among three groups. **a** MPV in patients with colorectal cancer or adenomatous polyp and healthy controls. **b** MPV/PC in patients with colorectal cancer or adenomatous polyp and healthy controls

The clinical characteristics of colorectal cancer, adenomatous polyp, and control groups are shown in Table 1. There was no significant difference in sex or age among the three groups. The values of MPV and MPV/PC in colorectal cancer, adenomatous polyp, and control groups are shown in Fig. 1. MPV was significantly lower in the colorectal cancer group than in the adenomatous polyp and control groups (cancer vs. adenomatous polyp, *p* = 0.002; cancer vs. control, *p* < 0.001). However, MPV did not significantly differ between the adenomatous polyp and control groups (*p* = 0.210). The MPV/PC was lower in the colorectal cancer group compared with the adenomatous polyp and control groups (both *p* < 0.001) and in the adenomatous polyp group compared with the control group (*p* = 0.010).

**Table 1:** Clinical characteristics of colorectal cancer, adenomatous polyp, and control groups

Parameters	Colorectal cancer group (*n* = 186)	Adenomatous polyp group (*n* = 132)	Control group (*n* = 108)	*p*-value
Age (years)	54.56 ± 12.18	52.55 ± 12.16	53.34 ± 14.18	0.369
Male (%)	111 (59.7%)	79 (59.8%)	65 (60.2%)	0.996
WBC (10^9^/L)	6.77 ± 1.64^a^	6.32 ± 1.61	6.23 ± 1.02^c^	0.003
RBC (10^12^/L)	4.42 ± 0.63^a^	4.78 ± 0.72	4.73 ± 0.42^c^	< 0.001
Hb (g/L)	121.27 ± 23.07^a^	132.12 ± 20.03^b^	142.47 ± 11.80^c^	< 0.001
Neutrophil (10^9^/L)	3.92 ± 1.26^a^	3.57 ± 1.26	3.40 ± 0.79^c^	< 0.001
Lymphocyte (10^9^/L)	1.99 ± 0.58	1.99 ± 0.60^b^	2.18 ± 0.51^c^	0.010
Monocyte (10^9^/L)	0.53 ± 0.19	0.50 ± 0.17^b^	0.45 ± 0.15^c^	0.001
Platelet count (10^9^/L)	279.80 ± 80.56^a^	223.89 ± 42.59^b^	207.83 ± 37.40^c^	< 0.001
MPV (fl)	8.48 ± 1.10^a^	8.83 ± 0.90	8.98 ± 0.77^c^	< 0.001
MPV/PC	0.0330 ± 0.0112^a^	0.0411 ± 0.0112^b^	0.0447 ± 0.0096^c^	< 0.001
NLR	1.98 (1.50–2.51)^a^	1.67 (1.37–2.27)^b^	1.51 (1.24–1.92)^c^	< 0.001
PLR	140.26 (107.11–182.96)^a^	113.03(90.98–143.81)^b^	94.55 (76.62–116.42)^c^	< 0.001

*WBC* white blood cells, *Hb* hemoglobin, *MPV* mean platelet volume, *MPV/PC* the ratio between MPV and platelet count, *NLR* neutrophil to lymphocyte ratio, *PLR* platelet to lymphocyte ratio

^a^Colorectal cancer group vs. adenomatous polyp group (*p* < 0.05)

^b^Adenomatous polyp group vs. control group (*p* < 0.05)

^c^Colorectal cancer group vs. control group (*p* < 0.05)

### Differences in MPV and MPV/PC among subgroups of colorectal cancer

The clinicopathological features of colorectal cancer subgroups and their preoperative values of MPV and MPV/PC are shown in Table 2. The MPV showed no significant differences among the subgroups regarding TNM stage, serosal invasion, lymph node metastasis, or distant metastasis. However, MPV/PC significantly differed between patients with stage I/II and stage III/IV cancer. Moreover, there was a significant difference in MPV/PC between patients with and without lymph node metastasis. The MPV/PC showed no significant differences among serosal invasion stages or between patients with and without distant metastasis.

**Table 2:** MPV and MPV/PC among subgroups of colorectal cancer patients

Parameters	*n*	MPV	*p*-value	MPV/PC	*p*-value
TNM stage					
I/II	92	8.37 ± 1.11	0.165	0.0311 ± 0.0089	0.021
III/IV	94	8.59 ± 1.08		0.0349 ± 0.0129	
Serosal invasion stage					
T1/T2	39	8.22 ± 0.99	0.092	0.0316 ± 0.0100	0.404
T3/T4	147	8.55 ± 1.11		0.0333 ± 0.0115	
Lymph node metastasis					
Absence	93	8.37 ± 1.10	0.164	0.0308 ± 0.0088	0.009
Presence	93	8.59 ± 1.08		0.0351 ± 0.0129	
Distant metastasis					
Absence	173	8.46 ± 1.08	0.306	0.0329 ± 0.0113	0.697
Presence	13	8.78 ± 1.25		0.0341 ± 0.0112	

*MPV* mean platelet volume, *MPV/PC* ratio between MPV and platelet count

### Correlations between MPV, MPV/PC and other hematological measures of inflammation

Correlation analysis demonstrated that the MPV negatively correlated with PLR (*r* = − 0.207, *p* < 0.001). However, the MPV did not correlate with NLR (*p* > 0.05).

Correlation analysis demonstrated that the MPV/PC was negatively correlated with the NLR (*r* = − 0.148, *p* = 0.002) and PLR concentration (*r* = − 0.575, *p* < 0.001).

### Diagnostic values of MPV, MPV/PC, NLR and PLR for differentiating between colorectal cancer and adenomatous polyp or controls

**Fig. 2:**
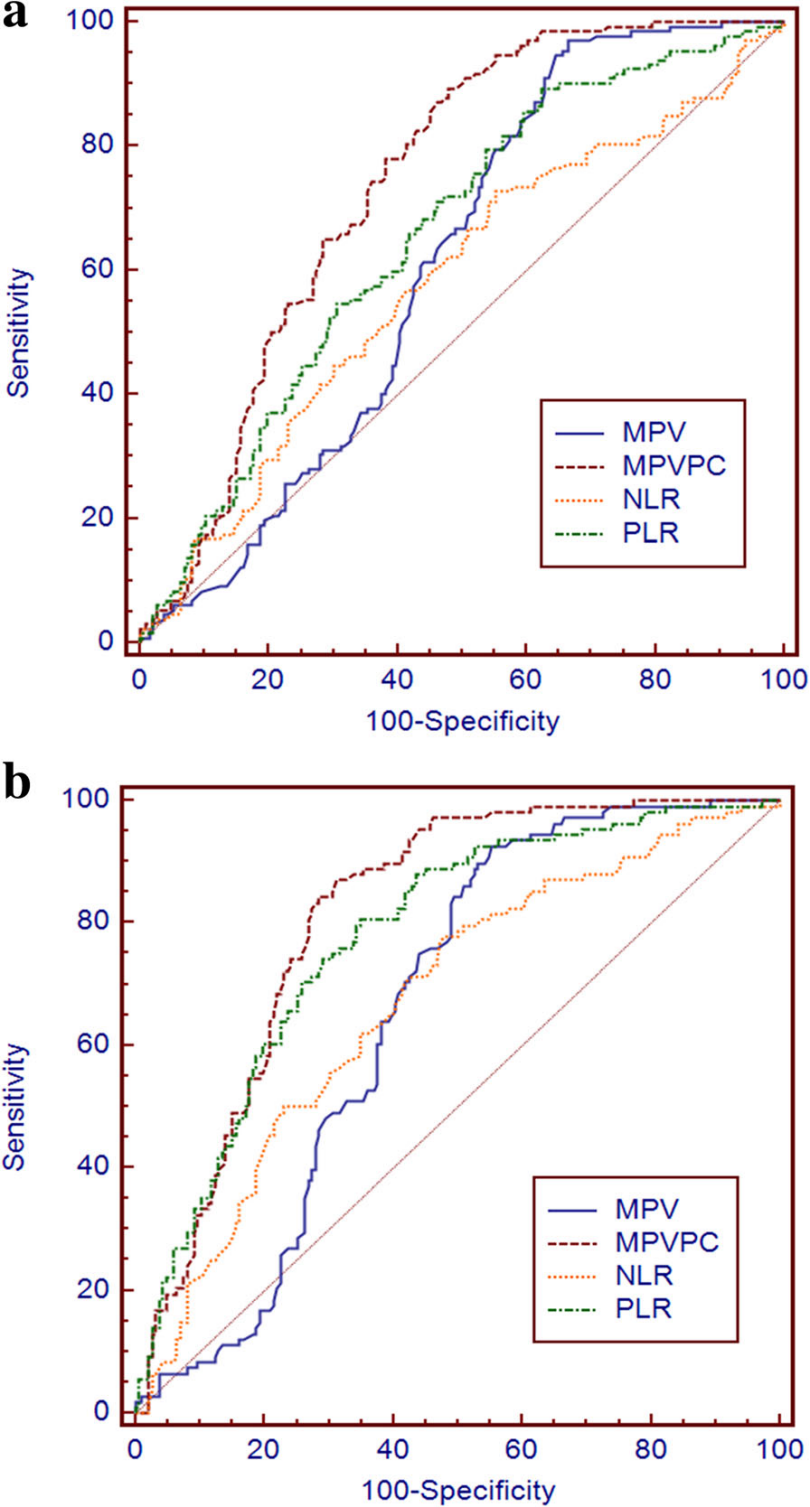
Diagnostic performances. **a** Diagnostic performances of MPV, MPV/PC, NLR, and PLR for distinguishing colorectal cancer from an adenomatous polyp. **b** Diagnostic performances of MPV, MPV/PC, NLR, and PLR for distinguishing colorectal cancer from healthy control

As shown in Table 3, with the adenomatous polyp as the reference, the specificity of MPV/PC was highest at 52.15 in colorectal cancer as compared with the adenomatous polyp. As shown in Table 4, with the controls as references, the specificity of MPV/PC was highest at 68.82 in colorectal cancer as compared with controls. Using MPV/PC to distinguish between colorectal cancer and adenomatous polyp produced a larger AUC than using MPV, NLR or PLR alone (all *p* < 0.05) (Fig. 2a). Using MPV/PC to distinguish between colorectal cancer and controls produced a larger AUC than using MPV or NLR alone (both *p* < 0.001) (Fig. 2b).

**Table 3:** Diagnostic performances of MPV, MPV/PC, NLR and PLR for distinguishing colorectal cancer from an adenomatous polyp

Marker	sensitivity	specificity	PPV	NPV	AUC (95% CI)
MPV	96.97	33.33	50.8	93.9	0.607(0.551–0.661)
MPV/PC	89.39	52.15	57.0	87.4	0.739(0.687–0.787)
NLR	72.73	44.62	48.2	69.7	0.579 (0.523–0.634)
PLR	89.39	37.63	50.4	83.3	0.654 (0.599–0.706)

*MPV* mean platelet volume, *MPV/PC* ratio between MPV and platelet count, *NLR* neutrophil to lymphocyte ratio, *PLR* platelet to lymphocyte ratio, *PPV* positive predictive value, *NPV* negative predictive value, *AUC* (95% CI) area under the receiver operating characteristic curve (95% confidence interval)

**Table 4:** Diagnostic performances of MPV, MPV/PC, NLR and PLR for distinguishing colorectal cancer from healthy controls

Marker	sensitivity	specificity	PPV	NPV	AUC (95% CI)
MPV	92.59	44.62	49.3	91.2	0.659(0.602–0.713)
MPV/PC	87.04	68.82	61.8	90.1	0.813(0.764–0.856)
NLR	77.78	52.15	48.6	80.2	0.673 (0.616–0.726)
PLR	80.56	65.05	57.2	85.2	0.777 (0.725–0.824)

*MPV* mean platelet volume, *MPV/PC* ratio between MPV and platelet count, *NLR* neutrophil to lymphocyte ratio, *PLR* platelet to lymphocyte ratio, *PPV* positive predictive value, *NPV* negative predictive value, *AUC* (95% CI) area under the receiver operating characteristic curve (95% confidence interval)

## Discussion

At present, the mechanistic relationship between MPV/PC and the occurrence or progression of malignant tumors remains unclear, but there are relatively more studies on MPV and PC separately than on MPV/PC. MPV represents the volume of platelets in the blood circulation and reflects their functional state [7, 13]. MPV is also an indicator of inflammatory diseases and is related to disease activity/severity [17, 18].

Studies have shown that inflammation is associated with the occurrence, development, and metastasis of multiple types of tumors [19–21]. The mechanisms may be related to the following reasons. First, inflammatory cells induce reactive oxygen species (ROS) production in cells, causing DNA damage and inhibiting DNA repair after injury, leading to tumorigenesis. Second, in the tumor microenvironment, inflammatory cells can secrete many cytokines, chemokines, and adhesion molecules in addition to promoting cell proliferation and angiogenesis, and these events promote tumor development and metastasis [22–24]. A decrease in MPV has also been implicated in locally advanced esophageal squamous cell carcinoma, gastric cancer, and bone marrow metastasis with solid tumors [25–27]. Platelets are non-nucleated cells that are produced by bone marrow megakaryocytes and are related to inflammation and thrombosis [28, 29]. Tumor cells associate with various cytokines such as platelet-derived growth factor, vascular endothelial growth factor, and other growth factors that stimulate platelet production [30]. Elevated platelet counts can produce more CD40L and promote an inflammatory response [29, 31, 32].Increased degradation of large platelets under inflammation may lead to a decrease in MPV, possibly because larger platelets are more responsive to stimulation, and a significant number of larger platelets are more likely to be selectively degraded [25].

In our study, the MPV values in the colorectal cancer group were significantly lower than those in the adenomatous polyp and control groups, which is in agreement with the findings reported by Cengiz et al. [33]. Sun et al. [25] found that MPV and MPV/PC were significantly decreased in patients with newly diagnosed locally advanced esophageal squamous cell carcinoma as compared to healthy controls. Inagaki et al. [34] found that the MPV and MPV/PC were significantly decreased in patients with non-small cell lung cancer as compared with controls. In our study, MPV/PC was significantly lower in the colorectal cancer group than in both the adenomatous polyp and control groups. Furthermore, MPV/PC negatively correlated with the NLR and PLR concentration. NLR and PLR are hematological measures of inflammation, which are related to several cancer types, including colorectal cancer, and renal cell carcinoma [35, 36]. Therefore, we speculate that the decrease of MPV/PC in colorectal cancer patients may be associated with an inflammatory reaction and suggest that it may be used as a marker to distinguish between benign and malignant colorectal tumors. MPV/PC is obtained by calculating the ratio of MPV to PC, which integrates the morphology and quantity of platelets and has a better diagnostic and predictive value than either parameter alone. The inverse relationship between MPV and PC may reflect the physiological tendency to maintain hemostasis [14, 37]. We also found that MPV showed no significant differences among subgroups regarding TNM stage, serosal invasion, lymph node metastasis or distant metastasis. The MPV/PC was significantly different in subgroups between patients with stage I/II and stage III/IV cancer. Moreover, there was a significant difference in MPV/PC between patients with and without lymph node metastasis. Therefore, we believe that MPV/PC may be useful for the differential diagnosis of early and advanced colorectal cancer.

In ROC curve analysis, a larger AUC indicates a better diagnostic efficiency. Cho et al. [14] found that the MPV/PC ratio showed a better result than MPV alone when using the AUC to evaluate the efficacy of MPV/PC for distinguishing between patients with hepatocellular carcinoma and healthy controls. Concordantly, when we used ROC curves to analyze the performance of MPV/PC for distinguishing colorectal cancer from benign colorectal polyps, MPV/PC showed superior diagnostic performance than using MPV, NLR or PLR alone. Using MPV/PC to distinguish between colorectal cancer and controls produced a larger AUC than using MPV or NLR alone.

Accordingly, we believe that MPV/PC may be a promising diagnostic biomarker for colorectal cancer.

This study had some limitations, as follows. First, relatively few cases were enrolled, and the conclusions need to be confirmed by large-scale multicenter clinical studies. Second, this study had a retrospective design, which cannot completely resolve some confounding factors and may produce a certain degree of deviation. Finally, we were not able to follow up patients with colorectal cancer and analyze the disease recurrence or the post-surgery status. Nevertheless, this study is the first to explore the relationship between MPV/PC and the clinicopathological features of colorectal cancer and assess its diagnostic value for colorectal cancer. The results highlight the possibility that MPV/PC could be used for the early detection and diagnosis of colorectal cancer.

## Conclusions

MPV/PC may be a useful diagnostic marker for distinguishing between benign and malignant colorectal tumors and between early and advanced colorectal cancer.

## Abbreviations

AUC (95% CI): Area under the receiver operating characteristic curve (95% confidence interval)
Hb: Hemoglobin
MPV: Mean platelet volume
MPV/PC: Ratio between MPV and platelet count
NLR: Neutrophil to lymphocyte ratio
NPV: Negative predictive value
PC: Platelet count
PLR: Platelet to lymphocyte ratio
PPV: Positive predictive value
ROC: Receiver operating characteristic
WBC: White blood cells
